# The clinical efficacy of pocket creation method ESD and conventional ESD in the treatment of early colorectal neoplasms: a meta-analysis

**DOI:** 10.3389/fmed.2026.1724880

**Published:** 2026-03-04

**Authors:** Kegumei Jiekang, Linyue Zhang, Geng Chen

**Affiliations:** Department of Gastroenterology, The First Hospital of Jilin University, Changchun, China

**Keywords:** conventional ESD, early colorectal neoplasms, endoscopic submucosal dissection, meta-analysis, pocket creation method

## Abstract

**Background:**

This study systematically evaluated the efficacy and safety of the pocket creation method endoscopic submucosal dissection (PCM-ESD) compared with conventional endoscopic submucosal dissection (C-ESD) in the treatment of early colorectal neoplasms.

**Methods:**

A comprehensive search was conducted across PubMed, The Cochrane Library, Embase, CNKI, VIP, and Wan Fang databases for all case–control studies published from the inception of these databases to June 30, 2025, comparing PCM-ESD with C-ESD in the treatment of early colorectal neoplasms. Literature screening and data extraction were performed in accordance with predefined inclusion and exclusion criteria. Meta-analysis was performed using RevMan 5.4 software to assess the efficacy and safety of both methods.

**Results:**

Nine studies, including 2 randomized controlled trials and 7 retrospective cohort studies with a total of 1,899 patients, were included. PCM-ESD achieved higher en bloc resection rates (RR = 1.05, 95%CI: 1.02 ~ 1.09, *p* = 0.004) and complete (R0) resection rates (OR = 2.34, 95% CI: 1.62–3.39, *p* < 00001) than C-ESD. Superior outcomes were also observed in fibrotic lesions (OR 1.67; 95% CI 1.14–2.43; *p* = 0.008) and LST-NG-type polyps (OR 1.39; 95% CI 1.09–1.79; *p* = 0.009). No significant differences were found in non-curative resection rates (OR 0.83; 95% CI 0.52–1.30; *p* = 0.41). PCM-ESD demonstrated shorter procedure time (min) (MD − 10.61; 95% CI − 15.41 to −5.80; *p* < 0.0001) and faster dissection speed (mm2/min) (MD 4.56; 95% CI 3.19–5.92; *p* < 0.00001). Perforation rates were lower with PCM-ESD (OR 0.41; 95% CI 0.20–0.81; *p* = 0.01), while bleeding rates were comparable (OR 1.03; 95% CI 0.51–2.05; *p* = 0.94). Specimen size was larger in the PCM-ESD group (MD 2.83; 95% CI 0.66–5.00; *p* = 0.01). Postoperative pathology revealed no difference in submucosal invasion depth (OR 0.94; 95% CI 0.69–1.28; *p* = 0.69).

**Conclusion:**

In the treatment of early colorectal neoplasms, PCM-ESD was associated with higher en bloc and R0 resection rates, shorter procedure time, faster dissection speed, and a lower perforation rate compared with C-ESD. In contrast, no statistically significant differences were observed between the two techniques with respect to non-curative resection rates, pathological outcomes, or bleeding-related events. While several efficacy and safety-related indicators favored PCM-ESD, these findings should be interpreted with caution given the predominance of retrospective data and the heterogeneity of study designs. Overall, PCM-ESD appears to be a promising alternative technique, particularly for technically challenging lesions.

**Systematic review registration:**

https://www.crd.york.ac.uk/prospero/, CRD420251147333.

## 1 Introduction

Colorectal cancer (CRC), as reported by the International Agency for Research on Cancer (IARC) under the World Health Organization, ranks as the third most common malignancy globally, with its incidence and mortality rates occupying the third and second positions, respectively, among all cancers. It represents one of the most prevalent malignant diseases of the digestive system (1, 2). In recent years, the incidence of CRC has continued to rise, largely driven by changes in lifestyle and environmental factors (3). In China alone, approximately 571,000 new cases and 240,000 colorectal cancers (CRC), accounting for the second and fourth highest rates of incidence and mortality nationwide (4). Early detection and intervention are critical to reducing colorectal neoplasms mortality (5). Currently, colonoscopy serves as both the primary diagnostic tool for early colorectal neoplasms and a crucial modality for therapeutic intervention. Among endoscopic therapeutic techniques, endoscopic mucosal resection (EMR) and endoscopic submucosal dissection (ESD) are most commonly used (6, 7). Both domestic and international clinical guidelines advocate for ESD as the preferred treatment for early-stage colorectal neoplasms. Compared with EMR, ESD achieves markedly higher en bloc resection rates, particularly for larger or laterally spreading lesions, and has therefore seen widespread clinical adoption. However, colorectal ESD faces significant technical challenges. These include the complex anatomy of the colon, interference from abdominal respiratory movements, and prolonged procedure durations, all of which elevate the technical difficulty and increase the likelihood of complications compared to its application in the upper gastrointestinal tract (8).

To address these limitations of conventional ESD (C-ESD), Hayashi et al. (9) introduced the Pocket Creation Method (PCM) in 2014. This technique is characterized by an initial minimal mucosal incision, followed by the systematic creation of a submucosal pocket beneath the lesion. The majority of submucosal dissection is performed within this pocket, after which a circumferential mucosal incision is completed. The procedure is typically facilitated by a small-caliber, tapered transparent cap, which provides both traction and counter-traction within the pocket, thereby improving visualization and stability of the operative field (10, 11). Compared with C-ESD, which entails an initial circumferential mucosal incision followed by stepwise submucosal dissection, this approach preserves and enhances submucosal tension, thereby markedly improving intraoperative visualization and procedural stability (12). In recent years, further refinements to PCM-ESD have been developed, including modified PCM techniques incorporating additional traction devices based on the original equipment (13, 14). In the present study, modified PCM-ESD and conventional PCM-ESD were not analyzed separately and were collectively categorized as PCM-ESD.

Preliminary studies have demonstrated that PCM-ESD offers advantages over C-ESD, particularly in resection rates and procedure times (15). This study aims to conduct a meta-analysis to compare the efficacy and safety of PCM-ESD versus C-ESD in the treatment of early colorectal neoplasms, with the goal of elucidating the clinical value of PCM-ESD.

## 2 Methods and materials

### 2.1 Search strategies

A comprehensive literature search was conducted in PubMed, The Cochrane Library, Embase, China National Knowledge Infrastructure (CNKI), VIP, and Wan Fang Data for case–control studies comparing PCM-ESD and C-ESD in the treatment of early colorectal neoplasms, published up to June 30, 2025. Literature screening and data extraction were performed in strict adherence to predefined inclusion and exclusion criteria, and the efficacy and safety of the two treatment modalities were subsequently analyzed. All data were processed using RevMan 5.4 software. Additionally, the references of the included studies were thoroughly reviewed, and corresponding authors were contacted via telephone or email for articles with unavailable full texts or incomplete data.

The search strategy incorporated a combination of subject headings and free-text terms. For Chinese databases, the following keywords were used: “endoscopic submucosal dissection,” “early colorectal neoplasms,” “early colorectal tumor,” “PCM-ESD,” “C-ESD,” and “ESD.” These terms were logically combined using the operators “OR” and “AND.” For English databases, search terms included “endoscopic submucosal dissection” or “ESD,” “early colorectal cancer” or “early colorectal tumor” or “early colorectal carcinoma,” “pocket-creation method ESD,” and “conventional method ESD,” also combined using “OR” and “AND.”

Literature screening and data extraction were carried out in strict accordance with predefined inclusion and exclusion criteria. Two independent reviewers evaluated the eligibility and methodological quality of each study. Disagreements were resolved through discussion or adjudication by a third reviewer. RevMan 5.4 software was used for data analysis. Reference lists of the included studies were manually screened to identify additional relevant articles. When full texts were unavailable or data were incomplete, corresponding authors were contacted via telephone or email:

1. Basic study information: Title, authors, publication date (year, volume, issue), etc.
2. Study design type.
3. Summary: Study location, sample size, patient demographics (age, gender ratio), and tumor characteristics (size, etc.).
4. Primary outcomes: en bloc resection rate, R0 resection rate, dissection speed, procedure time, bleeding, and perforation rates.
5. Secondary outcomes: non-curative resection rate, resection rate of LST-NG-type colorectal polyps, size of resected specimens, right colon lesion resection rate, resection rate of fibrotic lesions, and detection of submucosal invasion or carcinoma.
6. Quality assessment: Cohort studies were evaluated using the Newcastle-Ottawa Scale (16), while the Cochrane risk-of-bias tool was used for randomized controlled trials (RCTs). The quality assessment of the included studies was independently performed by two reviewers (Supplementary files 1, 2).

### 2.2 Study selection

Studies meeting all predefined inclusion criteria were incorporated into the analysis, whereas any study fulfilling one or more exclusion criteria was excluded.

#### 2.2.1 Inclusion criteria

1. Patients with early colorectal neoplasms eligible for ESD, including colorectal adenomas and early-stage colorectal carcinoma, as defined by endoscopic and histopathological criteria.
2. Cohort studies or randomized controlled trials (RCTs) that provided extractable outcome data relevant to ESD efficacy and safety, including but not limited to en bloc resection rate, R0 resection rate, procedure time, dissection speed, and procedure-related adverse events.
3. Studies in which PCM-ESD was used as the experimental intervention, with C-ESD serving as the control.

#### 2.2.2 Exclusion criteria

1. Studies reporting only the treatment outcomes of PCM-ESD or C-ESD individually.
2. Animal studies, case reports, systematic reviews, conference abstracts, and other non-primary research types.
3. Studies with fewer than 20 cases in either the experimental or control group.

#### 2.2.3 Outcome measures

En bloc resection rate, R0 resection rate, non-curative resection rate, dissection speed, procedure time, bleeding and perforation rates, LST-NG polypectomy rate, Size of resected specimens, resection rate of fibrotic lesions, pathologic findings. Procedure time (min) was measured from the commencement of mucosal incision to the completion of lesion excision. Dissection speed (mm^2^/min) was determined by dividing the resected specimen area (mm^2^) by the procedure duration (min).

### 2.3 Statistical methods

Meta-analysis was performed using RevMan 5.4 software. Continuous variables (e.g., procedure time, dissection speed, and specimen size) were analyzed using mean differences (MD) with 95% confidence intervals (95% CI). Dichotomous variables (e.g., en bloc resection, R0 resection, bleeding, perforation, and fibrosis) were analyzed using odds ratios (ORs) or risk ratios (RRs), together with corresponding 95% confidence intervals (CIs), as measures of effect size. For studies that reported only medians and ranges, means and standard deviations were derived based on established methodologies. Statistical significance was set at *α* = 0.05, with *p* < 0.05 indicating a significant difference (17–20).

#### 2.3.1 Heterogeneity and sensitivity analysis

Two independent researchers performed literature retrieval and screening according to predefined criteria. Heterogeneity across studies was assessed using the Q test and the *I*^2^ statistic. If *p* ≥ 0.1 and *I*^2^ < 50%, heterogeneity was deemed acceptable and a fixed-effect model was applied. If *p* < 0.1 or *I*^2^ ≥ 50%, substantial heterogeneity was assumed, and subgroup analysis, sensitivity analysis, or meta-regression was conducted to explore its sources. A fixed-effect model was used if the heterogeneity could be resolved; otherwise, a random-effects model was employed. Sensitivity analysis was carried out by sequentially removing individual studies and reanalyzing the pooled results. This approach was used to determine the influence of each study on effect estimates and to assess the robustness and stability of the findings (17–20).

## 3 Results

### 3.1 Literature selection and study characteristics

**Figure 1:**
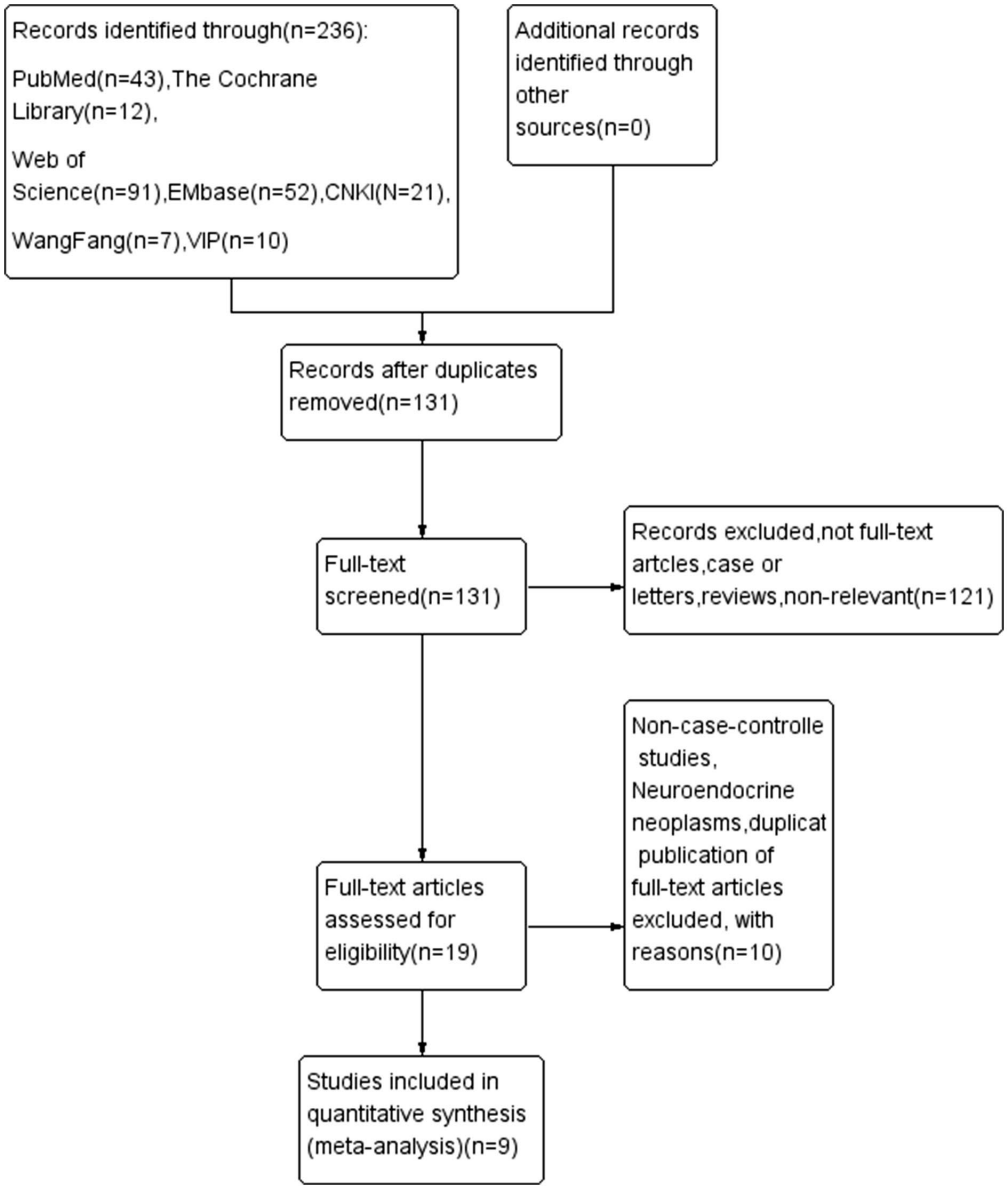
Flowchart summary of study identification and selection.

A total of 236 potentially relevant studies were initially identified. After screening titles, abstracts, and full texts, studies were excluded based on the following criteria: duplicate publications, reviews, commentaries, animal studies, interventions not meeting the specified criteria, studies with incomplete data, and studies with inaccessible full texts. The study by Sakamoto et al. (21) was excluded as its data were ultimately incorporated in the study by Takezawa et al. (12). In total, 9 studies were included in the final analysis, of which 2 were randomized controlled trials (RCTs) (13, 22) and 7 were cohort studies (12, 14, 23–27). All included studies, except for the one by Yang Dong et al. (27), were published in Japan between 2017 and 2022. The patient characteristics across the studies were summarized and analyzed, revealing no significant differences between the PCM-ESD and C-ESD groups with respect to gender, age, tumor size, or lesion location, as shown in Table 1. A total of 645 lesions were resected in the PCM-ESD group, and 1,254 lesions were resected in the C-ESD group, resulting in 1,899 lesions in total. Notably, in the study by Harada et al. (13), the modified PCM-ESD was referred to as the saline-pocket method of ESD (SP-ESD), in which normal saline was continuously injected into the submucosal pocket during dissection. The comparison between PCM-ESD and SP-ESD has not been previously addressed, and for this meta-analysis, no distinction was made between the two techniques. The literature screening process is illustrated in the PRISMA-P flowchart, as shown in Figure 1.

**Table 1:** Characteristics of enrolled patients and colorectal lesions.

Reference	Study type	Country	Patients/Lesions	Sex (male/ female)	Method	Age, years (range)	Location of lesion (n) (right/left/rectum)	Size (range) (mm)	En bloc resection (%)	R0 resection (%)
Takezawa et al. (12)	Retrospective	Japan	517/543	299/218	PCM	67 ± 9.9	215/65/0	35.3 ± 13.6	100	91
					CM	67 ± 10.0	200/63/0	35.7 ± 16.2	96	85
Harada et al. (13)	RCT	Japan	91	58/37	CM	71 (61–80)	25/17/6	25 (21.8–29.3)	100	100
					PCM	73 (64.5–76)	25/17/5	25 (21–29)	100	100
Naohisa et al. (24)	Retrospective	Japan	120	65/55	PCM	66.7 ± 10.5	12/0/9	30.1 ± 9.5 (18–50)	95.2	85.7
					CM	70.2 ± 10.8	43/20/36	34.5 ± 16.5 (10–70)	74.7	54.5
Naohisa et al. (24)	Retrospective	Japan	537	300/237	PCM	65.2 ± 13.5	16/8/13	31.1 ± 19.3 (10–60)	100	100
					CM	67.6 ± 10.6	270/85/144	37.3 ± 15.3 (10–140)	94.4	75.8
Akira et al. (23)	Retrospective	Japan	96	65/31	PCM	70 (41–83)	25/12/10	26 (20–68)	100	100
					CM	71 (44–83)	30/8/11	30 (20–58)	88	84
Daisuke et al. (14)	Retrospective	Japan	324	170/154	CE	65.0 (58–73.5)	88/36/63	25.0 (17.0–34.5)	97	91
					CM	65.0 (56.0–73.0)	46/20/12	30.0 (20.0–35.0)	97	91
					PCM	71.0 (62.5–77.5)	42/13/4	25.0 (20.0–30.0)	100	95
Ide et al. (26)	Retrospective	Japan	72	40/32	CM	66 (57–73)	13/7/21	No	78	66
					PCM	70 (60–73.8)	15/9/7	No	100	97
Yang et al. (27)	Retrospective	China	122	61/61	PCM	62.36 ± 9.32	36/0/0	24.7 ± 7.5	No	No
					CM	59.91 ± 9.79	86/0/0	21.1 ± 8.2	No	No
Yamashina et al. (22)	RCT	Japan	114	67/47	PCM	70 (41–92)	36/12/10	30 (18–75)	100	94
					CM	68 (42–86)	36/9/12	30 (20–80)	93	87
Yamashina et al. (25)	Retrospective	Japan	89/90	40/49	PCM	64.5 (42–90)	15/6/19	30.5 (20–57)	100	88
					CM	67 (42–88)	18/11/21	30.5 (20–90)	94	78

Two reviewers independently evaluated the methodological quality of the included studies. Cohort studies were assessed using the Newcastle–Ottawa Scale (NOS), with a maximum of 9 points: scores of 0–3 indicated low quality, 4–6 indicated moderate quality, and 7–9 indicated high quality. Randomized controlled trials (RCTs) were evaluated using the Cochrane Risk of Bias Tool. The results of the quality assessments are summarized in the corresponding figure (Supplementary files 1, 2).

### 3.2 Subgroup analysis

**Figure 2:**
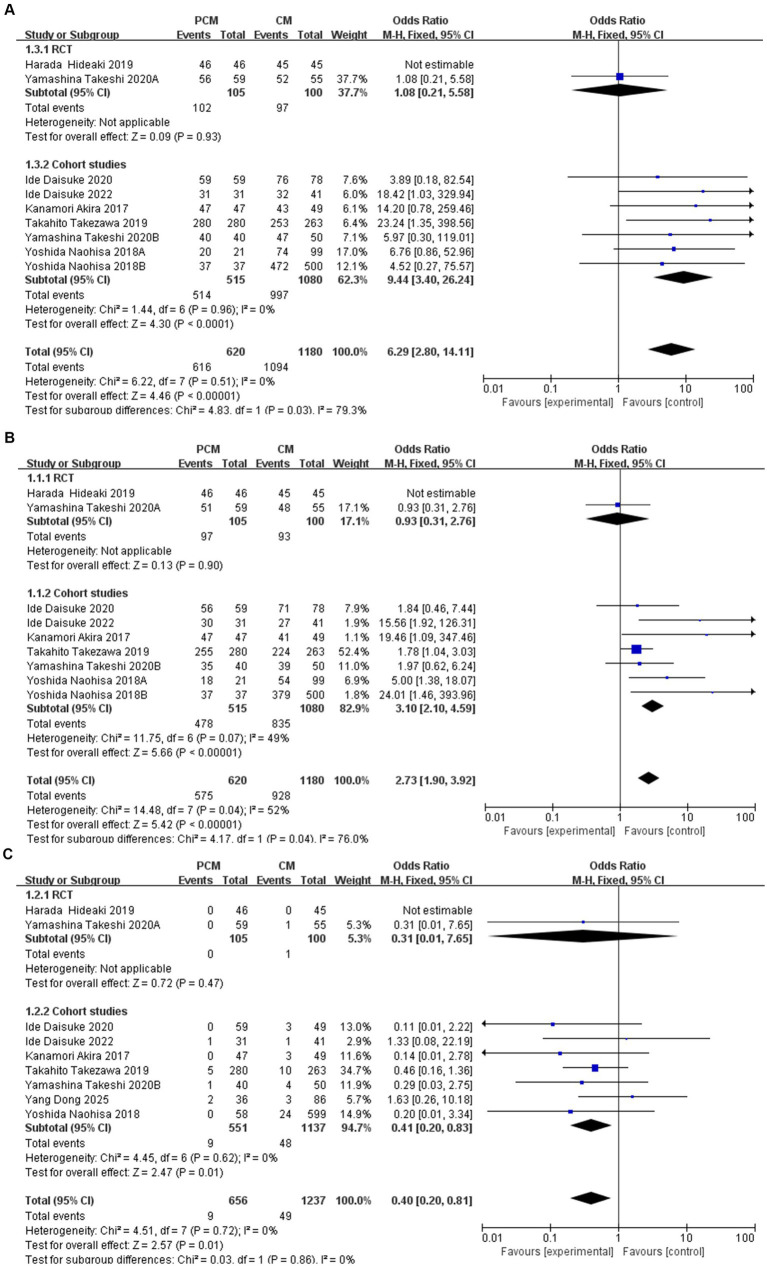
**(A)** En bloc resection rate by study design (RCTs vs. cohort studies). **(B)** R0 resection rate by study design (RCTs vs. cohort studies). **(C)** Perforation rate by study design (RCTs vs. cohort studies).

To examine the potential influence of study design on effect estimates, subgroup analyses were conducted separating RCTs from cohort studies. For en bloc resection, two RCTs showed no significant difference between PCM-ESD and CM-ESD (OR = 1.08, 95% CI: 0.21–5.58), whereas six cohort studies demonstrated a clear advantage of PCM-ESD (OR = 9.44, 95% CI: 3.40–26.24); the overall pooled analysis still indicated a significant benefit of PCM-ESD (OR = 6.29, 95% CI: 2.80–14.11), as shown in Figure 2A. Regarding R0 resection, RCTs again showed no significant difference (OR = 0.93, 95% CI: 0.31–2.76), while cohort studies revealed a marked improvement with PCM-ESD (OR = 3.10, 95% CI: 2.10–4.59); the pooled analysis continued to support the superiority of PCM-ESD (OR = 2.73, 95% CI: 1.90–3.92), as shown in Figure 2B. For perforation rates, RCTs showed no significant difference (OR = 0.31, 95% CI: 0.01–7.65), whereas cohort studies indicated a significant reduction in risk with PCM-ESD (OR = 0.41, 95% CI: 0.20–0.83), and the overall pooled trend was consistent (OR = 0.40, 95% CI: 0.20–0.81), as shown in Figure 2C. The pooled analysis showed a statistically significant association favoring PCM-ESD; however, this effect was primarily driven by cohort studies, whereas RCTs did not demonstrate a significant difference. Notably, effect estimates differed substantially by study design across all three outcomes, with RCTs consistently showing neutral results and cohort studies indicating larger effect sizes.

### 3.3 Publication bias assessment

**Figure 3:**
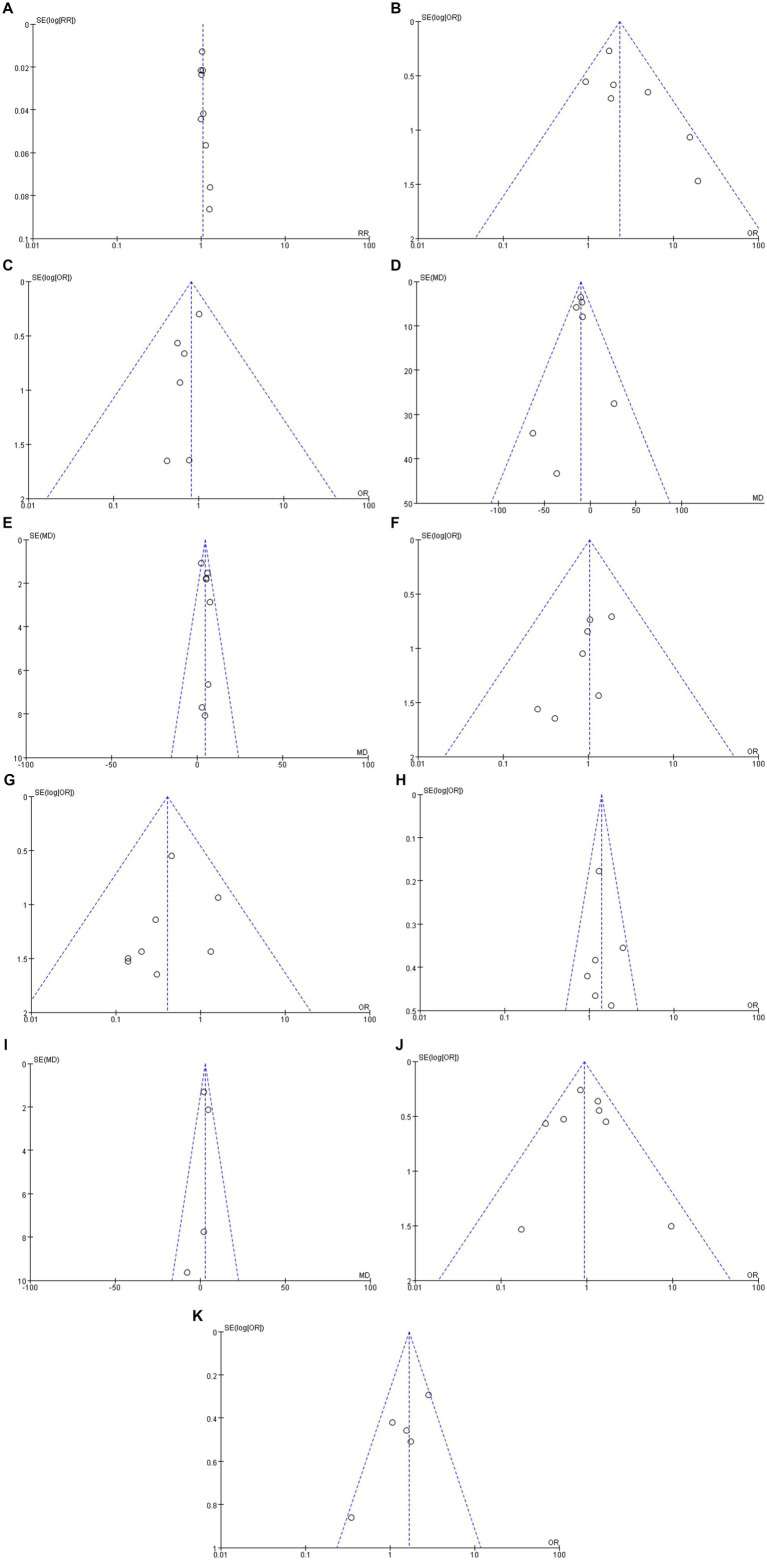
**(A)** En bloc resection rate. **(B)** R0 resection rate. **(C)** Non-tissue curative resection rate. **(D)** Procedure time. **(E)** Dissection speed. **(F)** Bleeding. **(G)** Perforation. **(H)** LST-NG type polyps. **(I)** Specimen size. **(J)** Pathologic findings. **(K)** Fibrotic lesions.

RevMan 5.4 software was used to synthesize the outcomes of the included studies and construct funnel plots to evaluate publication bias. A symmetrical funnel plot was interpreted as indicating no evidence of publication bias, whereas noticeable asymmetry suggested potential bias in the reported outcomes. The funnel plot used to assess publication bias is shown in Figures 3A-K.

### 3.4 Results

#### 3.4.1 En bloc resection rate

**Figure 4:**
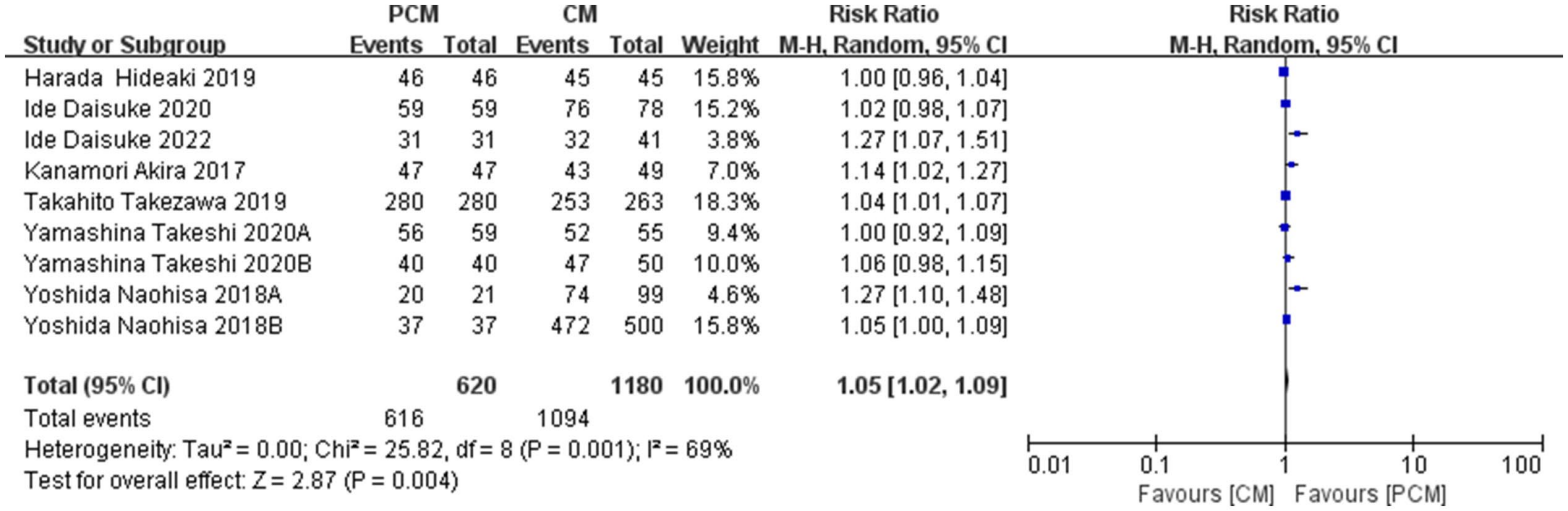
Comparison of *en bloc* resection rate between PCM-ESD and C-ESD.

A total of 8 studies (*N* = 1800) were included. Heterogeneity testing revealed no significant variability among the studies (*I*^2^ = 69%, *p* = 0.001). The fixed-effect model demonstrated that the PCM-ESD technique achieved a significantly higher En bloc resection rate compared to the C-ESD method (RR = 1.05, 95%CI: 1.02 ~ 1.09, *p* = 0.004) (Figure 4).

#### 3.4.2 R0 resection rate

**Figure 5:**
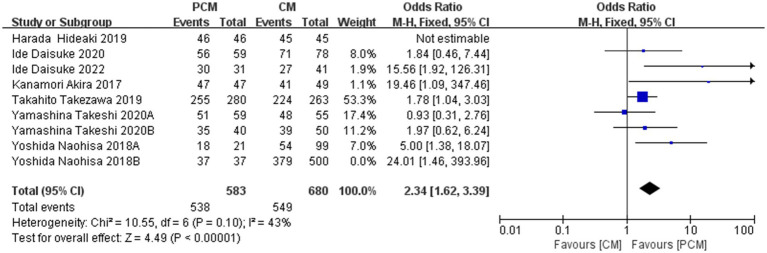
Comparison of the R0 resection rate between PCM-ESD and C-ESD.

A total of 8 studies (*N* = 1800) were included in the analysis. Heterogeneity testing indicated moderate variability among the studies (*I*^2^ = 52%, *p* = 0.04). Sensitivity analysis revealed that the data from Group B of Yoshida Naohisa et al.’s study were the primary source of heterogeneity. Upon exclusion of this study, heterogeneity decreased to an acceptable level (*I*^2^ = 43%, *p* = 0.1). Meta-analysis using a fixed-effect model demonstrated that the PCM-ESD group had a significantly higher R0 resection rate compared to the C-ESD group (OR = 2.34, 95% CI: 1.62–3.39, *p* < 00001) (Figure 5).

#### 3.4.3 Non-tissue curative resection rate

**Figure 6:**
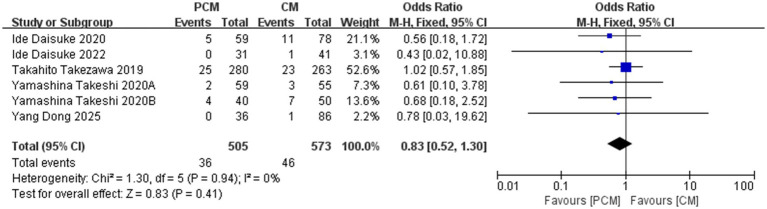
Comparison of non-organ curative resection rate between PCM-ESD and C-ESD.

A total of 6 studies (*N* = 1,078) were included in the analysis of the non-tissue curative resection rate. Heterogeneity testing revealed no significant variability among the studies (*I*^2^ = 0%, *p* = 0.94). The fixed-effect model indicated no significant difference in the non-tissue curative resection rate between PCM-ESD and C-ESD (OR = 0.83, 95% CI: 0.52–1.30, *p* = 0.41) (Figure 6).

#### 3.4.4 Procedure time

**Figure 7:**
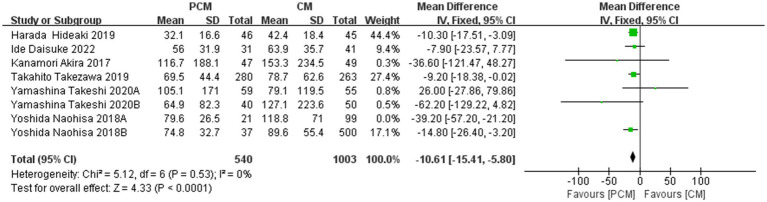
Comparison of the procedure time (minutes) between PCM-ESD and C-ESD.

A total of 7 studies (*N* = 1,543) were included in the analysis. Heterogeneity testing revealed significant variability among the studies (*I*^2^ = 0%, *p* = 0.53). Sensitivity analysis revealed that the data from Group B of Yoshida Naohisa et al.’s study were the primary source of heterogeneity. Upon exclusion of this study, heterogeneity decreased to an acceptable level (*I*^2^ = 0%, *p* = 0.53). The fixed-effect model demonstrated that the PCM-ESD group had a significantly shorter procedure time compared to the C-ESD group (MD = −10.61, 95% CI: −15.41 to −5.80, *p* < 0.0001) (Figure 7).

#### 3.4.5 Dissection speed

**Figure 8:**
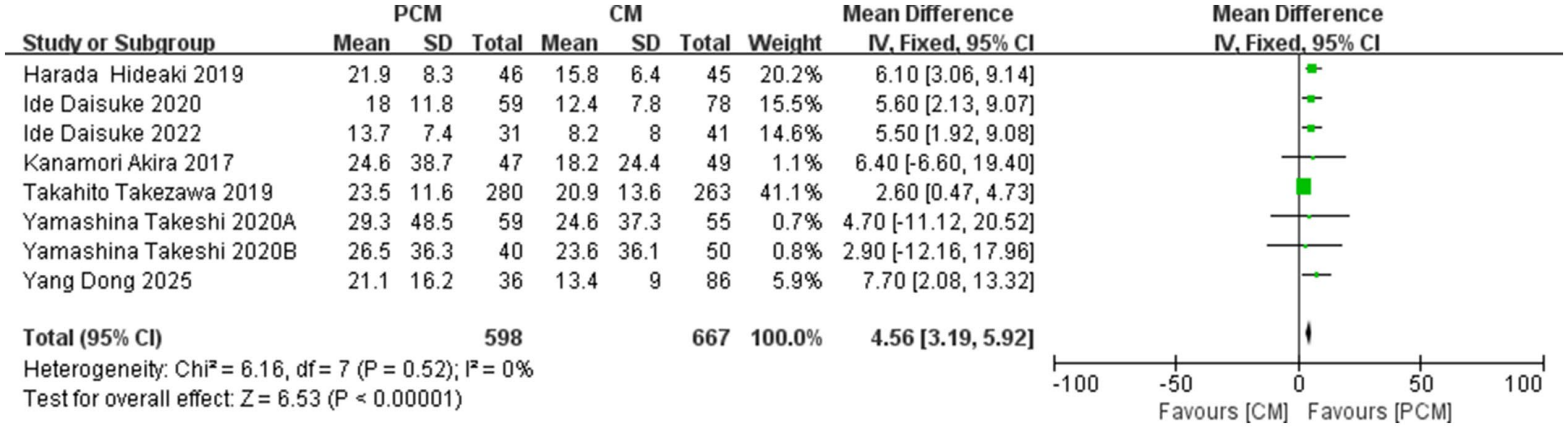
Comparison of the dissection speed (mm^2^/min) between PCM-ESD and C-ESD.

A total of 8 studies (*N* = 1,265) were included in the analysis of dissection speed. Heterogeneity testing revealed no significant variability among the studies (*I*^2^ = 0%, *p* = 0.52). The fixed-effect model yielded a statistically significant result (MD = 4.56, 95% CI: 3.19–5.92, *p* < 0.00001), indicating that the PCM-ESD group had a significantly faster dissection speed compared to the C-ESD group (Figure 8).

#### 3.4.6 Bleeding rate

**Figure 9:**
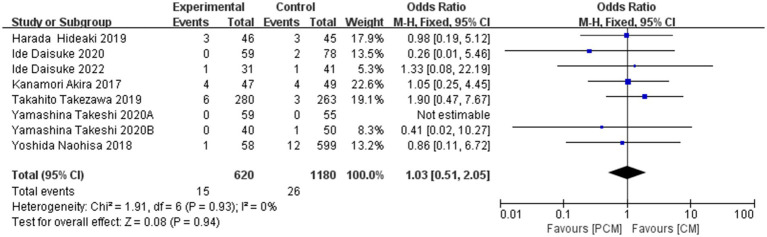
Comparison of the bleeding rate between PCM-ESD and C-ESD.

A total of 8 studies (*N* = 1800) were included in the analysis. The bleeding rate was 2.42% (15/620) in the PCM-ESD group and 2.2% (15/1180) in the C-ESD group. Heterogeneity testing indicated no significant variability among the studies (*I*^2^ = 0%, *p* = 0.93). Meta-analysis revealed no significant difference in the bleeding rate between the two groups (OR = 1.03, 95% CI: 0.51–2.05, *p* = 0.94). Sensitivity analysis demonstrated minimal fluctuation in the pooled OR following the exclusion of individual studies. Additionally, the funnel plot did not suggest significant publication bias, confirming the stability and reliability of the meta-analysis results. In conclusion, there was no significant difference in the incidence of bleeding complications between PCM-ESD and C-ESD (Figure 9).

#### 3.4.7 Perforation rate

**Figure 10:**
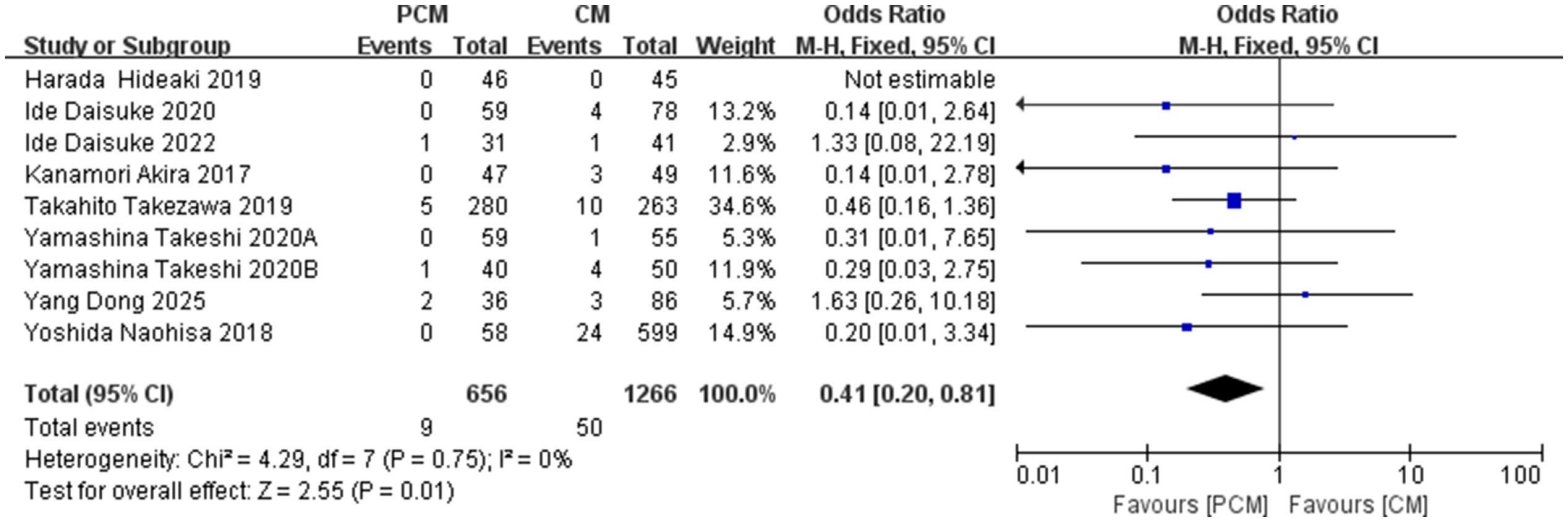
Comparison of the perforation rate between PCM-ESD and C-ESD.

A total of 9 studies (*N* = 1922) were included in the analysis of perforation rates. The perforation rate was 1.37% (9/656) in the PCM-ESD group and 3.94% (50/1266) in the C-ESD group. Heterogeneity testing revealed no significant variability among the studies (*I*^2^ = 0%, *p* = 0.75). The fixed-effect model demonstrated that the perforation rate in the PCM-ESD group was significantly lower than that in the C-ESD group (OR = 0.41, 95% CI: 0.20–0.81, *p* = 0.01) (Figure 10).

#### 3.4.8 The LST-NG

**Figure 11:**
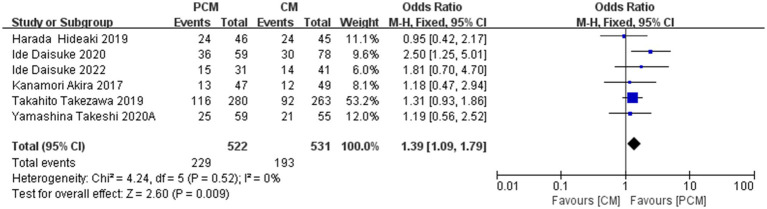
Comparison of the LST-NG between PCM-ESD and C-ESD.

A total of 6 studies (*N* = 1,053) examining LST-NG colorectal polyps were included. Heterogeneity testing revealed no significant variability among the studies (*I*^2^ = 0%, *p* = 0.54). The fixed-effect model demonstrated that the PCM-ESD group had a significantly higher resection rate for LST-NG polyps compared to the C-ESD group (OR = 1.39, 95% CI: 1.09–1.79, *p* = 0.009) (Figure 11).

#### 3.4.9 Size of specimen

**Figure 12:**
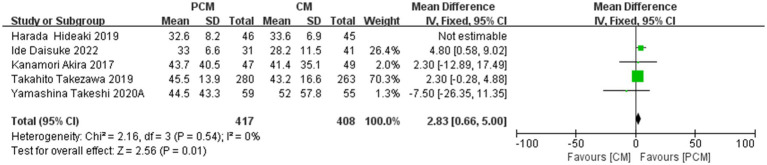
Comparison of the size of specimen between PCM-ESD and C-ESD.

A total of 5 studies (*N* = 916) were included in the analysis of resection specimen size. Heterogeneity testing revealed no significant variability among the studies (*I*^2^ = 34%, *p* = 0.19). The fixed-effect model indicated a statistical result of MD = 2.83, 95% CI: 0.66–5.00, *p* = 0.08. While no significant heterogeneity was observed, sensitivity analysis revealed a considerable change in the effect size after excluding the study by Harada Hideaki et al., suggesting potential publication bias. After excluding this study, 4 studies (*N* = 825) remained, and the fixed-effect model demonstrated that the PCM-ESD group had a significantly larger resection specimen compared to the C-ESD group (MD = 2.83, 95% CI: 0.66–5.00, *p* = 0.01) (Figure 12).

#### 3.4.10 Postoperative pathologic findings

**Figure 13:**
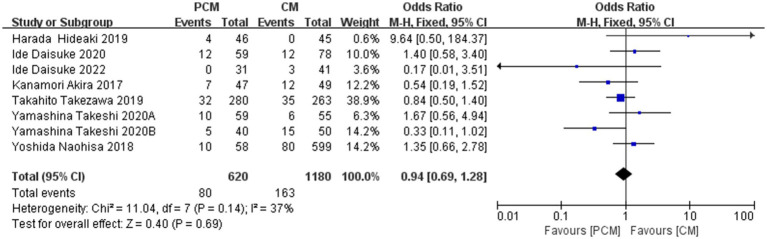
Comparison of the pathologic findings between PCM-ESD and C-ESD.

A total of 8 studies (*N* = 1800) with postoperative pathology confirming submucosal invasion or advanced colorectal cancer were included. Heterogeneity testing indicated no significant variability among the studies (*I*^2^ = 37%, *p* = 0.14). The fixed-effect model demonstrated that no statistically significant difference between the two surgical methods (OR = 0.94, 95% CI: 0.69–1.28, *p* = 0.69). Sensitivity analysis and the final funnel plot demonstrated minimal fluctuation in the OR values, suggesting the robustness and reliability of the meta-analysis results. There was no significant difference in the detection rate of submucosal invasion or advanced cancer in early colorectal cancer between PCM-ESD and C-ESD (Figure 13).

#### 3.4.11 Fibrotic lesions

**Figure 14:**
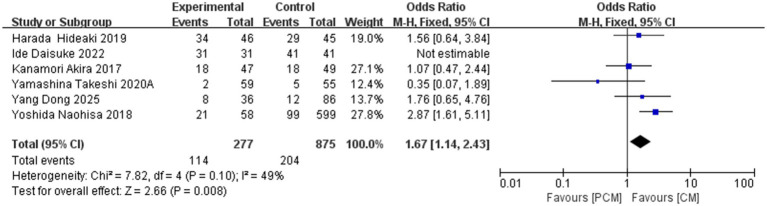
Comparison of the fibrotic lesions between PCM-ESD and C-ESD.

A total of 6 studies (*N* = 1,152) on fibrotic lesions were included. Heterogeneity testing revealed no significant variability among the studies (*I*^2^ = 49%, p = 0.1). The fixed-effect model demonstrated that the PCM-ESD group had a significantly higher resection rate for early colorectal cancer with fibrotic lesions compared to the C-ESD group (OR = 1.67, 95% CI: 1.14–2.43, *p* = 0.008) (Figure 14).

## 4 Discussion

This meta-analysis systematically evaluates the efficacy and safety of PCM-ESD compared to C-ESD in the treatment of early colorectal neoplasms. The pooled results from the included studies demonstrate that PCM-ESD is associated with improvements in several key procedural outcomes, including resection quality and procedural efficiency, as well as a reduced risk of perforation. However, for other clinically relevant endpoints, such as bleeding events and pathologic findings, no statistically significant differences were observed between the two techniques, aligning with findings from recent studies (12–14, 21–27).

As endoscopic techniques have evolved, ESD has become the primary modality for treating early colorectal neoplasms. However, challenges such as the complex anatomy of the colon, thin colonic walls, and operational interference from respiratory movements significantly increase the procedural difficulty. Additional factors, including fibrotic lesions, large lesions size, and limited operator experience, contribute to higher rates of incomplete resection, bleeding, and perforation. To address these challenges, Hayashi et al. introduced the PCM in 2014, a novel ESD technique designed to improve surgical efficacy and reduce complications. PCM-ESD offers four distinct advantages (12, 28): (1) Enhanced operational control: By creating a submucosal pocket through a minimal mucosal incision, PCM stabilizes the tip of the endoscope, ensuring consistent positioning within the submucosal layer. (2) A small-Caliber tapered transparent cap within the submucosal pocket allows for optimal dissection angles, improving visualization of the muscular layer and preserving submucosal integrity, thus reducing perforation risks. (3) The submucosal pocket ensures prolonged mucosal lifting, preventing rapid dispersion of injected fluid, reducing the need for repeated injections, and minimizing thermal damage during the procedure. (4) The tapered transparent cap provides effective traction, mitigating the technical challenges posed by the colonic anatomy and respiratory movements.

This meta-analysis included studies covering a variety of early colorectal neoplasms, including large laterally spreading tumors, recurrent lesions, and fibrotic lesions. Comparative analysis revealed significant advantages of PCM-ESD in both en bloc resection rates and R0 resection rates. Notably, Takezawa et al. (12) reported a 100% en bloc resection rate for PCM-ESD, significantly higher than the 96% achieved with C-ESD (*p* < 0.001). The R0 resection rate was also markedly superior (91% vs. 85%, *p* = 0.033), with the advantage particularly pronounced in recurrent lesions with severe fibrosis. In a 2018 study, Yoshida et al. (24) reported that PCM-ESD maintained higher en bloc and R0 resection rates in lesions with severe fibrosis compared to C-ESD. Ide et al. (14) further combined PCM-ESD with a traction device, achieving a 100% en bloc resection rate for fibrotic lesions. This suggests that PCM-ESD effectively avoids positive vertical margins, enhancing the accuracy of pathological assessments. Moreover, the present meta-analysis demonstrates that PCM-ESD significantly shortens procedural time and accelerates dissection speed, thereby markedly improving overall procedural efficiency. Consistent with these findings, the study by Harada *et al.* reported a substantially higher dissection speed with PCM-ESD compared with C-ESD (20.1 vs. 16.3 mm^2^/min, *p* < 0.001), accompanied by a significantly reduced procedure time (29.5 vs. 41 min, *p* < 0.001). Additionally, Yang et al. (27) showed that PCM-ESD is particularly advantageous for lesions located in the cecum and ascending colon, where the thin wall, deep location, and respiratory motion make dissection difficult. It should be noted that, in the present analysis, PCM-ESD was not explicitly distinguished from PCM-ESD with traction. As traction devices are independently known to improve visualization, tissue exposure, and procedural control during ESD, part of the observed procedural advantages in the PCM-ESD group—particularly in studies incorporating adjunct traction—may be attributable to the effect of traction itself rather than to PCM-ESD alone. Accordingly, the pooled estimates should be interpreted with caution, and the independent contribution of PCM-ESD warrants further investigation.

From a safety perspective, perforation remains one of the most severe complications of ESD. If endoscopic closure fails, surgical intervention is often required, resulting in poor prognosis and elevated treatment costs (29). Multiple studies in this meta-analysis indicated that PCM-ESD not only enhances surgical efficiency but also ensures higher safety. Yamashina et al. (25) studied large non-pedunculated colorectal lesions (≥20 mm) and found that, despite the larger size and presence of fibrosis, the incidence of severe complications such as bleeding and perforation was comparable to or lower than that seen with C-ESD. For lesions with high malignant potential, such as LST-NG (30), and those with submucosal fibrosis due to prior biopsy or inflammation, C-ESD often faces technical challenges, including poor surgical field visibility, increased dissection difficulty, high perforation risk, and higher rates of incomplete resection. Our analysis indicates that PCM-ESD outperforms C-ESD in terms of applicability and effectiveness in managing these complex lesions, offering a more reliable clinical solution. Moreover, Yoshida et al. (24) emphasized that PCM-ESD, unlike C-ESD, does not form flaps that obstruct subsequent operations, making it particularly advantageous for giant lesions (≥50 mm in diameter). PCM-ESD also lowers the technical threshold, allowing less experienced endoscopists to efficiently and successfully perform the procedure.

In 2020, Ide et al. (14) proposed traction device-assisted PCM-ESD (PCM with TD), which facilitates easier creation of mucosal flaps and faster formation of submucosal pockets. This method was shown to be more efficient for technically challenging lesions, with non-expert endoscopists achieving results comparable to those of experts. Takezawa et al. (12) also demonstrated that the proportion of expert operators was significantly lower in the PCM-ESD group (28.9%) than in the C-ESD group (43.3%) (*p* < 0.001), while en bloc and R0 resection rates were still significantly higher in the PCM-ESD group.

PCM-ESD, as an innovative derivative of C-ESD, demonstrates substantial advantages over standard circumferential incision ESD in terms of overall therapeutic efficacy and safety, particularly for lesions with complex anatomical locations, severe fibrosis, or diameters exceeding 20 mm. However, this meta-analysis highlights that the specimens obtained via PCM-ESD tend to be larger than those from C-ESD. For early colorectal neoplasms with smaller lesion sizes or simpler morphological features (e.g., non-fibrotic, superficial, or flat benign lesions), clinicians should carefully assess the appropriateness of PCM-ESD to avoid unnecessary complexity. In such cases, C-ESD or other simpler endoscopic techniques may provide a more favorable balance between efficiency and clinical benefit, while PCM-ESD should be reserved for more challenging cases.

This study has several limitations. First, the meta-analysis included only nine studies, seven of which were cohort studies and only two randomized controlled trials (RCTs), resulting in a relatively small sample size. Secondly, subgroup analyses demonstrated that while cohort studies consistently suggested a significant advantage of PCM-ESD in terms of en bloc resection, R0 resection, and perforation rates, RCTs did not demonstrate statistically significant differences between PCM-ESD and CM-ESD. This discrepancy may be explained by the inherent limitations of observational studies, including residual confounding, selection bias, and learning-curve effects, which are difficult to fully adjust for despite methodological rigor. Therefore, although the pooled estimates suggest a potential benefit of PCM-ESD, these results should be interpreted with caution. The absence of a clear advantage in randomized evidence indicates that the true clinical effect of PCM-ESD may be smaller than that suggested by observational data. Thirdly, most studies were conducted in a single country or region, which introduces geographical limitations. Therefore, the results should be interpreted with caution, and further large-sample, high-quality prospective RCTs are necessary to validate these findings.

## Data Availability

The datasets presented in this study can be found in online repositories. The names of the repository/repositories and accession number(s) can be found in the article/Supplementary material.

## Supplementary material

SUPPLEMENTARY FILE 1 Summary of risk bias in randomized controlled trial.

SUPPLEMENTARY FILE 2 NOS for Cohort studies Quantitative Scoring.
