# Supplementary figures and images for: The clinical efficacy of pocket creation method ESD and conventional ESD in the treatment of early colorectal neoplasms: a meta-analysis
Source: Front Med (Lausanne). 2026 Mar 4;13:1724880. doi: 10.3389/fmed.2026.1724880 (PMC12997784; doi:10.3389/fmed.2026.1724880)

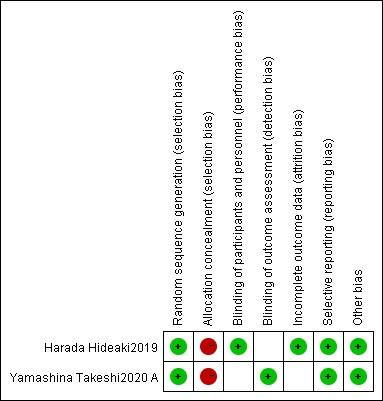

Supplement: SUPPLEMENTARY FILE 1 — Summary of risk bias in randomized controlled trial. [file Supplementary_file_1.tiff]
