# Supplementary material for: The clinical efficacy of pocket creation method ESD and conventional ESD in the treatment of early colorectal neoplasms: a meta-analysis
Source: Front Med (Lausanne). 2026 Mar 4;13:1724880. doi: 10.3389/fmed.2026.1724880 (PMC12997784; doi:10.3389/fmed.2026.1724880)
Supplement: SUPPLEMENTARY FILE 2 — NOS for Cohort studies Quantitative Scoring. [file Supplementary_file_2.docx]

| Reference | Time |  | Selection | | | Comparability | Outcome | Follow-up adequacy | Follow-up  completeness | Total score |
| --- | --- | --- | --- | --- | --- | --- | --- | --- | --- | --- |
|  |  | S  1 | S  2 | S  3 | S  4 |  |  |  |  |  |
| Takahito Takezawa | 2019 | 1 | 1 | 1 | 1 | 1 | 1 | 0 | 0 | 6 |
| Yoshida Naohisa | 2018 | 1 | 1 | 1 | 1 | 1 | 1 | 0 | 0 | 6 |
| Kanamori Akira | 2017 | 1 | 1 | 1 | 1 | 1 | 1 | 0 | 0 | 6 |
| Ide Daisuke | 2022 | 1 | 1 | 1 | 1 | 1 | 1 | 0 | 0 | 6 |
| Ide Daisuke | 2021 | 1 | 1 | 1 | 1 | 2 | 1 | 0 | 0 | 7 |
| Yang Dong | 2025 | 1 | 1 | 1 | 1 | 1 | 1 | 0 | 0 | 6 |
| Yamashina  Takeshi B | 2020 | 1 | 1 | 1 | 1 | 2 | 1 | 0 | 0 | 7 |

Supplement2: NOS for Cohort studies Quantitative Scoring
